# A Mixture of “Cheats” and “Co-Operators” Can Enable Maximal Group Benefit

**DOI:** 10.1371/journal.pbio.1000486

**Published:** 2010-09-14

**Authors:** R. Craig MacLean, Ayari Fuentes-Hernandez, Duncan Greig, Laurence D. Hurst, Ivana Gudelj

**Affiliations:** 1 Department of Zoology, University of Oxford, Oxford, United Kingdom; 2 Department of Biology and Biochemistry, University of Bath, Bath, United Kingdom; 3 Department of Mathematics, Imperial College London, London, United Kingdom; 4 Research Department of Genetics, Evolution, and Environment, University College London, London, United Kingdom; 5 Max Planck Institute for Evolutionary Biology, Plön, Germany; University of Lausanne, Switzerland

## Abstract

Is a group best off if everyone co-operates? Theory often considers this to be so (e.g. the “conspiracy of doves”), this understanding underpinning social and economic policy. We observe, however, that after competition between “cheat” and “co-operator” strains of yeast, population fitness is maximized under co-existence. To address whether this might just be a peculiarity of our experimental system or a result with broader applicability, we assemble, benchmark, dissect, and test a systems model. This reveals the conditions necessary to recover the unexpected result. These are 3-fold: (a) that resources are used inefficiently when they are abundant, (b) that the amount of co-operation needed cannot be accurately assessed, and (c) the population is structured, such that co-operators receive more of the resource than the cheats. Relaxing any of the assumptions can lead to population fitness being maximized when cheats are absent, which we experimentally demonstrate. These three conditions will often be relevant, and hence in order to understand the trajectory of social interactions, understanding the dynamics of the efficiency of resource utilization and accuracy of information will be necessary.

## Abstract 2

It is commonly assumed that the world would be best off if everyone co-operates. Experimental and mathematical analysis of “co-operation” in yeast show why this isn't always the case.

## Author Summary

The world is best off, it is usually presumed, when everyone co-operates. However, we discovered in a laboratory experiment involving yeasts that a population can grow more and faster when there is a mix of “cheats” and “co-operators.” In this case “co-operator” cells produce a protein (invertase) that breaks down sugar in the environment enabling it to be used by anyone. “Cheats” eat the broken down sugar but don't produce invertase and so have fewer costs. How can it be that yeast populations do best when such apparently selfish cheats are common? To resolve this we constructed a mathematical model, used this to discover reasons why the classical result wasn't found, and experimentally verified these conclusions. We find three conditions required to recover the unexpected result: (1) the “co-operators” should get more food than “cheats” (e.g. if the two aren't perfectly mixed together), (2) food is used more efficiently when there is a famine than when there is a feast, and (3) the amount of “co-operation” given should not accurately match the amount needed. We argue that all three are likely not to be peculiar to yeast, suggesting that “cheats” may be good for a group in many cases.

## Introduction

Wild caught strains of yeast are polymorphic [1] for the ability to produce the enzyme invertase. Strains with *SUC2* secrete the enzyme, which catalyses the hydrolysis of sucrose into glucose and fructose. These are transported into the cell by hexose transporters and metabolized through glycolysis [2]. By contrast, *suc2* strains do not secrete invertase and, as a consequence, do not suffer the manufacturing costs. Nonetheless, they consume the glucose and fructose. Both strains can also metabolize sucrose, taking it up through an active sucrose-H^+^ symport [3]–[5], but metabolism of glucose is more efficient and preferred [6]. Those strains that secrete invertase are considered “co-operators,” while non-producers are regarded as selfish “cheats” [2],[7],[8].

The competition between these two strains has been configured as a snowdrift game [8], a sub-class of public goods game [7]. The snowdrift game [9] envisages two parties stuck in a snowdrift that need to clear the snow (hydrolyze sucrose) to be able to move on (grow). A co-operator helps shift the snow (makes invertase), while a defector doesn't. There exists a benefit to clearing the way (making glucose available) and a cost to shoveling snow (the cost of invertase). In its simplest form, we suppose the benefit to clearing the snow is *b*, the cost to removing all of the snow is *c*. A co-operator playing against a co-operator thus gains benefit *b* while suffering the cost *c*/2, with net effect *R* = *b*−*c*/2. A cheat playing a co-operator gains the benefit *b* with net effect *T* = *b*, while the co-operator has net effect *S* = *b*−*c*. Two defectors playing each other gain no benefit and suffer no cost with net effect *P* = 0. Snowdrift dynamics require that *T*>*R*>*S*>*P*. Under these circumstances, the population payoff, assuming random encounters, is:
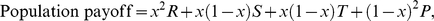
where *x* is the frequency of co-operators. Population payoff is maximal when:
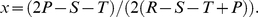
Incorporating the terms of cost and benefit, population fitness is maximal when all co-operate (*x* = 1). In this and related co-operation games in the economic, social, and evolutionary sciences, it is thus classically supposed (either explicitly or as a necessary consequence of assumed pay-offs) [10]–[12], and sometimes experimentally reported [13]–[15], that population fitness is maximized when cheats are absent. This understanding is encapsulated in the concept of the “conspiracy of doves,” the idea that in the hawk-dove game (a manifestation of the snowdrift game [16]), the population would be best off if all played the more cooperative non-aggressive dove strategy [17]. The same notion is commonly core to policy efforts aimed at maximization of co-operation and to modeling efforts aimed at understanding the dynamics of co-operation.

The conspiracy of doves, while a commonly assumed notion, is, we note, not a necessary assumption. One can, in principle, consider versions of the snowdrift game in which the co-occurrence of cheats and co-operators maximizes population fitness. In examining competition between our two strains we indeed discovered that population net growth was not maximal when non-producers, the putative “cheats,” are absent. While this is at odds with a considerable body of prior co-operation theory, it is also necessary to ask whether what we have discovered has relevance beyond our system and, if so, under what conditions? To establish the underlying causes of the unexpected result, and in turn to understand whether it is likely to be just a curiosity of our system, we construct a systems model of the interaction. Our approach is to start by specifying a relatively complex and highly parameterized model that can capture experimental results. This we benchmark by reference to experimental results. We then attempt to modify the model across multiple parameters, so as to identify the necessary conditions for the recovery of the novel result, as opposed to the classical result (maximal fitness when the population consists exclusively of co-operators). We then experimentally confirm these conditions, where possible. Before this we determine whether the interaction could be fairly considered a “cheat-co-operator” system.

## Results

### Invertase Production Is Costly But Can Increase Fitness

If invertase secretion is a co-operative trait, we would expect that invertase secretion increases the average fitness of the group at a direct cost to individuals that secrete the enzyme. To test the hypothesis of a direct cost, we competed a producer strain of yeast that carries a single active *SUC2* gene against an isogenic non-producer mutant that refrains from invertase secretion (*suc2*). We make use of the fact that invertase production is conditional on extra-cellular glucose levels [18]. By performing the competition in a glucose-limited chemostat (see Methods: Experimental Design A), we can thus induce invertase secretion, without any possible benefit of invertase secretion, as no sucrose is present. In this experiment the *suc2* mutant strain enjoys a 4% fitness advantage (w = 1.04, s.e. 0.014, *n* = 15, t_14_ = 2.72, *p* = 0.016). We conclude that invertase manufacture and secretion can be costly.

To test the hypothesis that invertase secretion increases mean fitness when sucrose is present, we assayed the pure culture growth rate of *SUC2* and *suc2* on agar plates containing sucrose (see Methods: Experimental Design B). Populations of producers have a maximal growth rate of 0.56 doublings per hour (s.e. = 0.002, *n* = 4), which is approximately 20% higher (t_6_ = 9.85, *p*<0.0001) than the growth rate of non-producers grown in isolation (0.46 doublings per hour, s.e. = 0.01, *n* = 4). As the glucose produced by producers is accessible by all cells [8], we conclude that invertase secretion can increase group fitness. Invertase production thus appears to conform to the assumptions of a co-operative trait as defined by social evolution theory.

### Population Fitness Is Maximal When Producers and Non-Producers Co-Exist

**Figure 1:**
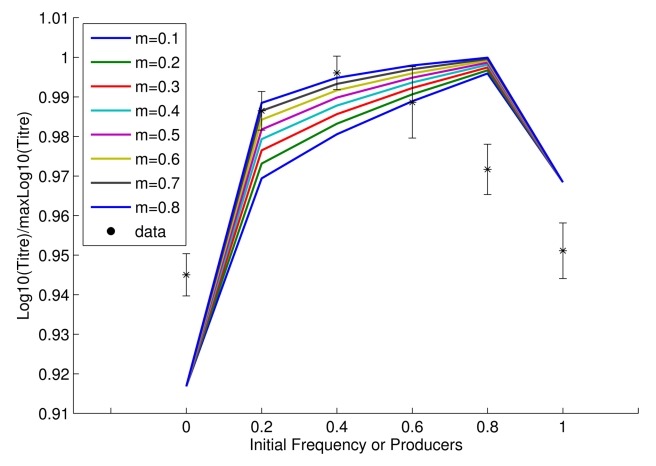
Final population size (Log(titre, normalized to maximum observed titre)) after exhaustion of resources as a function of initial invertase producer frequency, in theory (lines) and practice (points (*); mean ± s.e.m.; *n* = 9). Observed data fit a quadratic function better than a linear function (F_2,3_, = 41.3, *p*<0.01).

**Figure 2:**
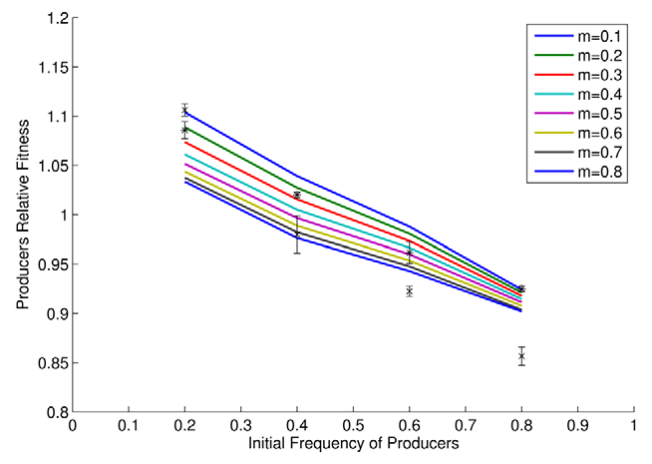
Relative producer fitness as a function of initial frequency in theory (lines) and practice (points (*); mean ± s.e.m.; *n* = 3). Asterisks represent poorly mixed cultures (*m* low) while data points marked with an x represent better mixed cultures (*m* high).

We established competition cultures of a *SUC2* strain and a *suc2* strain that were grown up overnight in YPD broth. Sucrose-limited 20 mL agar plates were inoculated with 20 20 mL aliquots of competition cultures (for more details see Methods: Experimental Designs C and D). Population fitness, measured as titre of cells after all sugar is exhausted, peaks when both producers and non-producers are present (Figure 1). This result suggests a new reason why a diversity of strategies is seen in social interactions. In such situations, in both nature and in humans, it is quite common [2],[7],[19]–[21] to observe the co-existence of apparent cheats and co-operators. This is also the case for invertase production: most strains of yeast secrete invertase, but approximately 10% of strains refrain [1]. Our results suggest that competition between groups (the net productivity effect that we observe) as well as within groups, mediated as negative frequency dependent selection (Figure 2), can both select for a diversity of strategies. The independence of the within- and between-group effects needs emphasis. In a snowdrift game formulation of the yeast system, for example, co-operators and cheats can be stably maintained even in approximately homogeneous environments [8]. In part this is because invertase is retained in the vicinity of *SUC2* strains, ensuring that producer strains receive a disproportionate amount of free glucose. This, however, is independent of any effect on population fitness, as the games predicting polymorphism also predict maximal population fitness when cheats are absent [8].

### A Systems Model

To investigate the unexpected behavior we start by assembling and validating a mathematical model of the condition, attempting where possible to respect the known biology of our experimental system.

#### Growth kinetics

In our model, both strains take up resources *R* and convert it into ATP using a simple, unbranched metabolic pathway (see e.g. [22]). The rate of ATP production in the pathway is denoted by *J^ATP^* and is given by:
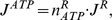
where *J^R^* denotes the rate of the pathway which is a function of resource concentration *R* and is mathematically represented as *J^R^*(*R*). The term 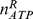 denotes the number of ATP molecules produced in the pathway. The yield of ATP production is known to depend on the rate of resource uptake, termed rate-yield trade-off; therefore, 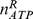 is a decreasing function of *J^R^*. In Bauchop and Elsden [23] it was observed that if microbes are limited by their energetic resource, the amount of biomass formed per unit of ATP is approximately constant and does not depend on the mode of ATP production. Therefore, as highlighted by Pfeiffer and Bonhoeffer [22], if the rate of ATP production increases, the rate of biomass formation and thus the growth rate of an organism also increases. This implies that the microbial growth rate can be represented as a linear function of the rate of ATP production, namely *r*⋅*J^ATP^*, where *r* is some proportionality constant. Here we take *r = 1*.

In practice yield of ATP production 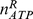 is not as easy to measure as the efficiency, 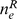 whereby
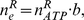
where *b* is a constant denoting the amount of biomass formed per unit of ATP. Therefore throughout the article, we consider rate-efficiency instead the rate-yield trade-off.

#### Sucrose utilization

Both yeast strains can take up sucrose (*S*) through an active sucrose-H^+^ symport, which is shown to be mediated by two different transport systems: high-affinity uptake mediated by *AGT1* permease and the low affinity pathway mediated by *MALx1* maltose transporters [5],[24]. Sucrose is a disaccharide and is transported into the cell slowly and inefficiently. The rate of this pathway is denoted by *J^S^* and its efficiency by 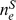.

Note that while yeast SUC knockout strains do not use internal invertase to hydrolyses sucrose, they can nonetheless metabolize it efficiently using internal maltase [25], this being the normal mode of sucrose utilization for yeast species lacking invertase [26]–[28]. A further possibility that we don't model, however, is that sucrose is hydrolyzed under acidic conditions outside of the cell in a non-enzymic process, with glucose and fructose then taken up in the normal manner. Given both that we employ a buffered medium and that the half-life of sucrose under our experimental conditions is 440 years [29], this doesn't seem especially likely. Several points of evidence support this supposition. Notably, *suc2Δ* strains without maltase cannot grow on sucrose [25], while *suc2Δ* missing the hexose import channels (necessary for glucose/fructose uptake from the exterior) grow well [4]. This suggests that internal maltase metabolized sucrose is needed, while external acid-hydrolyzed sucrose is not sufficient to support the growth we observe of non-producers.

#### Invertase production

Invertase producers secrete invertase, which catalyzes the hydrolysis of sucrose (*S*) into glucose (*G*) and fructose (*F*), monosaccharides which are transported into the cell [30]. The rate of conversion of sucrose into glucose and fructose (*Inv*) is assumed to have the following form:
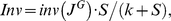
where *inv* denotes invertase activity, which is known to be a function of glucose consumption rate [18]. Here we also assume that the rate of sucrose degradation is a saturating function of sucrose concentration with *k* denoting a saturation constant and we take *k = 10^−4^*. Note that invertase production is conditioned not on sucrose levels (as would seem optimal) but on local glucose levels, high glucose suppressing invertase production, an absence of glucose resulting in a residual low level production and medium levels stimulating invertase.

Invertase is costly to produce and the cost function (*c^Inv^*) varies with glucose level in the following way *c^Inv^ = inv(* *J* *^G^)U^Inv^*, where *U^Inv^* denotes the unit cost of invertase, which is a function of invertase activity. As invertase activity increases we assume that production per unit invertase becomes more costly as every invertase molecule made means one molecule of some other important protein is not made [31],[32].

#### Glucose and fructose utilization

Glucose and fructose are transported into the cell by hexose transporters. We assume that there is one non-specific site available for glucose and fructose to bind. Yeast utilizes glucose as a preferential carbon source and the preferential uptake of glucose over fructose [33]–[35] is modeled as competition for this site in the following way. The rate of the hexose pathway when glucose is transported is defined by
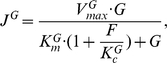
where the rate of the same pathway when fructose is transported is defined by
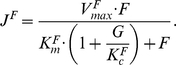
Here 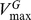 (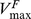) denotes the maximal rate of the pathway for glucose (fructose), while 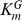 and 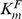 denote the respective Michaelis-Menten constants. The preferential uptake of glucose over fructose [33]–[35] we model as competition for this site using competition constants 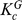 and 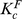. When there is no competition both *F/* 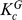 *= 0* and *G*/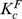 = 0 and the classical Michaelis-Menten kinetics are recovered.

The pathway rate represents the rate at which product is formed, which in this case is the same as the rate at which substrate is consumed. Therefore throughout this article we refer to 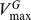 (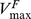) as the maximal rate of glucose (fructose) uptake and 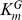 (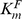) as the measure of affinity for glucose (fructose). The efficiency of the pathway utilizing glucose and fructose is denoted by 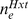, which is a function of both glucose and fructose uptake rate, and hence we write 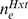 *(* *J* *^G^*+*J* *^F^)*. Yeast exposed to abundant hexose convert it inefficiently into growth compared with those exposed to lower hexose levels [36],[37]. We term this a rate-efficiency trade-off, where an increase in resource uptake rate leads to a decrease in the number of cells created per unit of resource, and therefore 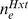 is a decreasing function of *J* *^G^*+*J* *^F^*. Note that ethanol production is negligible and is not considered when modeling hexose metabolism.

To predict densities of the co-operator/producer (*N*_p_) and cheat/non-producer (*N*_n_) strains in a well-mixed environment, we then use equation:
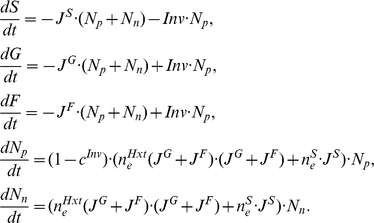
(1)In addition, to model our experimental setup, we consider both the initial spatial structure of otherwise immotile yeast cells as well as the movement of sugars by diffusion. The spatial structure is modeled phenomenologically using the mixture parameter 0≤*m*≤1. This parameter captures the extent to which cells of a given type have a different type as a possible neighbor, such that when *m = 0* the two strains are spatially segregated, while when *m = 1* the two strains are perfectly well-mixed. For 0<*m*<1 the environment can be approximately considered as if consisting of three different regions: region 1, where producers are surrounded only by their own type; region 2, where non-producers are surrounded only by their own type; and region 3, where producers and non-producers are neighbors. The proportion of all cells in region 3 approximates to *m*. Our two mixing regimes on agar plates do not have precise representations as regards the parameter *m* but can be considered *m* high or *m* low.

As resources diffuse through the environment, the spatial structure of the population is not alone enough to reflect the spatial distribution of resources. In our model the “movement” of resources is dependent on the diffusion rate, *D*, which reflects the rate at which resources available to one strain become available to the other by moving through regions 1, 2, and 3. Note that secreted invertase remains localized between the cell membrane and the cell wall, and therefore the enzyme itself does not diffuse. This leads to the expansion of the model (1) into
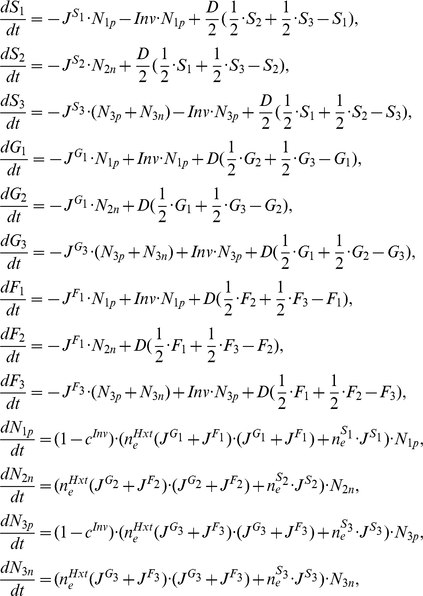
(2)where the subscript *i* = 1, 2, 3 denotes the spatial region *i* discussed above.

The model was parameterized where possible using known biochemical rate constants. Where the form of a curve was known but exact parameters unknown, these were estimated from simple growth experiments (see Supplementary Methods 1 in Text S1). For each simulation we consider the same starting concentration of sucrose *S_in_* (2% unless stated otherwise) and the same starting number of cells *N* *_in_* but vary both the ratio of producers to non-producers *(f)* and the degree of mixing *(m)*. This leads to the following initial conditions:
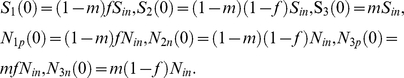
The simulations are run until resources are exhausted. This is the relevant termination condition both for our circumstance and also more generally for consideration of finite resources. The model does not hence ask about evolutionary stability, as evolutionarily stable conditions need not be found prior to the exhaustion of a limited resource (what may be considered immediate population stability, in the sense that the population size is fixed and cannot grow). The model can, however, predict when the starting frequencies of producers and non-producers are the same (i.e., when relative fitness = 1) and hence when stable equilibrium will be seen after multiple iterations of seasonality.

### Benchmarking the Model

The model captures the population fitness maximization when non-producers are present (Figure 1), a property that to the best of our knowledge is unique to this model. Note too that the model was not constructed in a manner designed to recover this result but rather to reflect known details of the biology and biochemistry of yeast. That such an “end-blind” model can capture unexpected experimental results suggests it to be fit for purpose.

This result is also, at least in theory, independent of the definition of population fitness. The population fitness we defined above as the total cell productivity after all sucrose is exhausted. This is equivalent to population fitness for *K* selected organisms. Were *r* selection more relevant, one might prefer to consider population growth rate (per unit time) instead. Using this definition of population fitness does not, at least in theory, importantly affect our conclusion that population fitness is maximal when producers and non-producers co-exist (Supplementary Result 1a). Model results for the total population growth are reported in the article and for population growth rate in Supporting Information (see Supplementary Results 1a–e).

We can further establish whether the model is fit for purpose by examining additional predictions. Our model, for example, predicts negative frequency dependence of relative fitness (Figure 2). Although in contrast to Hamilton's theoretical result [38] that inclusive fitness of co-operators is not a function of co-operator frequency, this result is not without precedent (e.g. [8],[39],[40]). Our competition experiments between isogenic *SUC2* and *suc2* knock-out strains of yeast confirm that selection for invertase production is indeed negatively frequency-dependent (Figure 2; F_1, 20_ = 290, *p*<1×10^−4^). Our model also predicts that increasing population structure modestly increases relative fitness of producers, as a higher proportion of glucose goes to the producers (note different intercepts of approximately parallel lines in Figure 2). This result is also confirmed by our experiments (Figure 2; F_1, 20_ = 13.96, *p* = 0.0013).

Given the observed negative frequency dependence, our model can also predict the stable equilibrium frequencies of producers and non-producers under different experimental regimes, these occurring when the relative fitnesses are the same. By fitting a quadratic function to the observed data in Figure 2, for high *m* the experimental equilibrium is estimated to be around 0.38 producers. The model predicts for *m* between 0.5 and 0.8 an equilibrium in the range of 0.31–0.39. For low *m* the observed equilibrium position is estimated to be around 0.46, the predicted range (*m* from 0.4–0.1) is between 0.42 and 0.55. We conclude that the model has a respectable ability to quantitatively predict equilibrium frequencies. At equilibrium the population is predicted to have higher fitness than a population of all producers. The model equilibrium frequencies are not the same as those that maximize population fitness. At equilibrium, there thus remains a conflict between individual and group “best interests”.

### Assumptions of Benefits and Costs and the Peculiar Behavior of Population Titre

Why does this model find that apparent cheats promote population growth where a prior snowdrift formulation did not (for comparison of this prior model and experimental results with ours see Supplementary Results 2) [8]? Might it be a consequence of features specific to yeast and incorporated in our model or might it be owing to factors that are likely to be more broadly applicable? To approach this we modify the model so as to determine the necessary conditions for the maintenance of the core result, namely that population growth is maximal in the presence of non-producers.

Our model makes assumptions about costs and benefits that are appropriate for our situation but that are typically not configured in the more general-purpose heuristic models of co-operation discussed above. We highlight two evident differences. First, snowdrift assumes the benefit to be fixed and constant, such that the *b* term is the same for all players gaining a benefit. More generally, game theoretical models usually presume that each unit of resource gained represents one unit of benefit. This is not true in yeast. While the growth rate is dependent on glucose concentrations, high local concentrations lead to inefficient utilization on a per molecule basis.

Similarly, the snowdrift game considers costs to be equally shared by all co-operators, that cost is linearly proportional to work done, and that there is a fixed total cost to removal of snow, this dictated by the amount of snow to be shoveled (e.g. co-operators stop shoveling when the road is cleared). Importantly, yeast are prone to violating this last assumption as they adjust their invertase production to the local glucose level, not to the sucrose level, ensuring a disconnect between the amount of “co-operation” needed (sucrose to be digested; snow to be shoveled) and the amount of “co-operation” offered (invertase production; snow shoveled).

**Figure 3:**
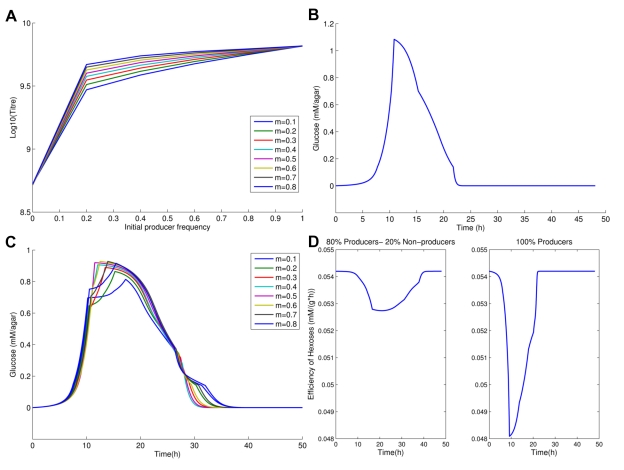
The role of the rate-efficiency trade-off and the dynamics of sugar metabolism. (a) Expected final population size (Log(titre)) after exhaustion of resources as a function of initial producer frequency in the absence of rate-efficiency trade-off. The temporal glucose spike, with glucose measured in mM/agar and time represented in hours, (b) when initially all the population are producers and (c) when 80% are producers with glucose measured in mM/agar and time is in hours. Note that the spike in (c) is lower and longer-lived, hence glucose is used more efficiently. (d) Efficiency of hexose usage by producers (g protein/mM hexose) when non-producers are present (80∶20 ratio: left hand panel) and when they are absent, i.e. 100% producers (right hand panel). Here we average across spatial structures.

Might modification of either of these biologically verified assumptions explain why non-producers stimulate population growth? Leaving the observed costs in place, we find that removal of the assumption of the rate-efficiency trade-off restores the usual assumption that population fitness is highest in the absence of cheats (Figure 3a). We can test the proposal that the rate-efficiency trade-off is important by making use of a particular feature of yeast's metabolism, namely that at very low sucrose levels the rate efficiency trade-off is very weak or non-existent [37]. We thus repeated our experiments at a very low sucrose level (0.01%) (Experimental Design E) and observe just the predicted behavior (Supplementary Results 3a). This does not, however, mean that the space in which maximal population fitness is associated with a mixture of producers and non-producers need be limited. If we consider an intermediate sucrose level, for example, we experimentally recover the humped distribution (Supplementary Results 3b), as predicted.

The rate-efficiency trade-off matters most if one considers the temporal trajectory of co-operation and population growth. When producers are especially common, the invertase production results in a large immediate spike, both spatial and temporal, in glucose. This would enable rapid but inefficient growth. If we replace a few producers with non-producers, the glucose spike would be smaller, so the population burns the finite resource more efficiently. The net effect then is to ensure sucrose is more efficiently converted to growth, but only if there is a rate-efficiency trade-off.

Consistent with this explanation, when producers are common, a high but short-lived temporal (and spatial) peak in free-glucose is observed in the model (Figure 3b), compared with the rather slower and more protracted production seen when producers are a little less common (Figure 3c). An even lower level of producers ensures, however, that internally metabolized sucrose is the predominant nutrient and this is also inefficient. As then expected, the efficiency (conversion of hexose to protein) of producers is radically degraded when the spike in glucose is observed, while a relatively small reduction is seen when cheats are present, even in an 80∶20 mix (Figure 3d).

**Figure 4:**
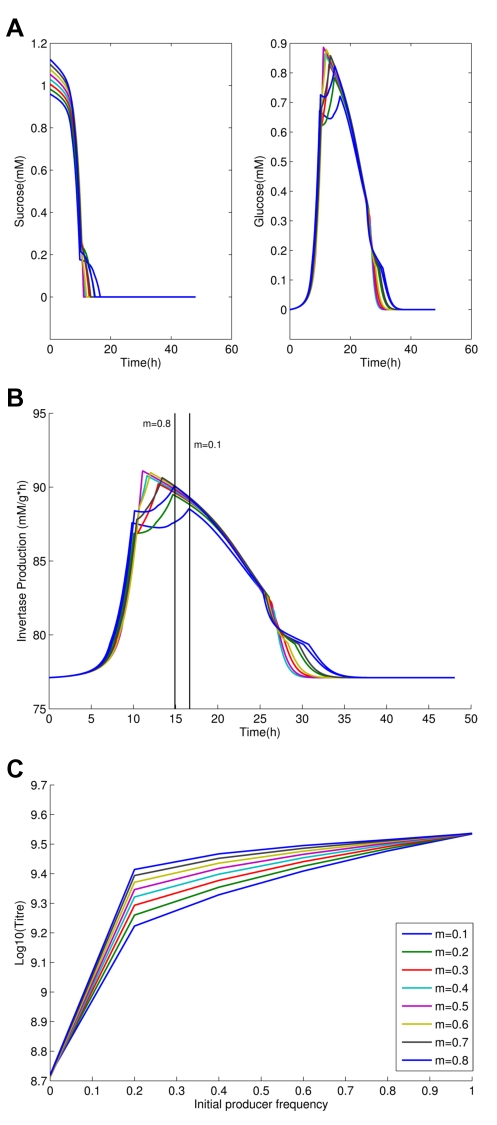
The importance of costly invertase production and its coupling with sucrose levels. (a) Sucrose and glucose levels (mM/agar) across the time course of the experiment (in the vicinity of region 3); (b) corresponding invertase production levels (mM glucose/g protein/hour); time of sucrose exhaustion is indicated by vertical black lines for m = 0.8 and m = 0.1. Note sucrose has disappeared relatively early but invertase is still produced thereafter; (c) expected final population size (Log(titre)) after exhaustion of resources as a function of initial co-operator frequency when cost of invertase production is reduced from 4% to 2% for invertase production of 86.7 mM glucose/g protein/hour that is 12% higher than the base-level invertase production.

From examination of the time course we also observe that sucrose is typically exhausted early on, but with invertase production being conditional on low glucose import rates, the producers make expensive, but useless, invertase through much of the latter part of the experiment (Figure 4a–b). To employ the metaphor of the snowdrift game, they are shoveling snow after the path is cleared. If invertase production is costly, producers thus retard population growth rates once all the sucrose has been hydrolyzed. We should then expect that the population titre peak is more likely to disappear as costs tend to zero. Indeed, we observe this in the model (Figure 4c).

**Figure 5:**
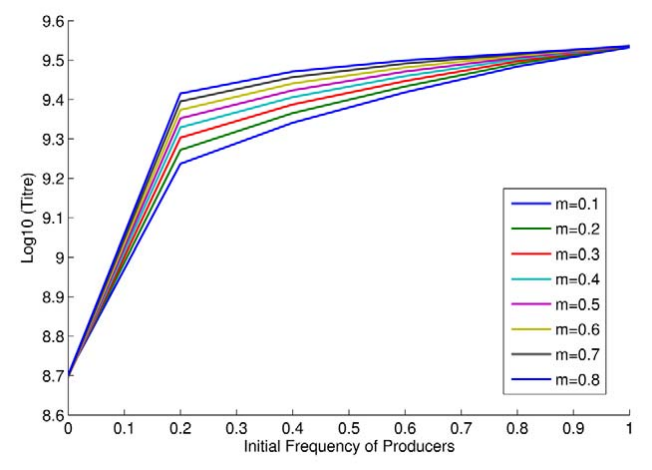
Theoretical expectations for titre when invertase production matches sucrose levels (perfect information).

Moreover, if yeast make invertase at a rate dependent upon the amount of sucrose available (and don't make invertase when sucrose is absent), we might also expect to find the classical result of maximum productivity when cheats are absent. To examine this we consider a model in which invertase production follows Michealis-Menten dynamics as a function of sucrose levels, rather than glucose levels, with zero production when sucrose is absent. This is equivalent to yeast having perfect information. As expected we find that, even with a rate-efficiency trade-off and costly invertase production, maximum population productivity occurs in the model when cheats are absent (Figure 5). Thus imperfect information can also yield the unexpected result. While we provide an experimental test of the other predictions of our model (see above and below) this one is not obviously amenable to experimental manipulation.

Aside from these two assumptions, we also model a spatially structured population. This is expected to be important as well-mixed populations share resources equally. To this end we can consider what happens when *m* = 1. When this occurs the model again recovers the classical result. This prediction we test by considering what happens in very well shaken flasks (Experimental Design F), this providing the best approximation of an absence of population structure. As predicted, in well-mixed populations there is no evidence (to any measureable degree) that population fitness is highest when producers and non-producers co-exist (Supplementary Results 4).

### The Economics of Inefficiency

The above demonstrates that three features are required to recover our non-classical result, that population fitness is maximal in the presence of non-producers. Modifications of some of these features can be seen as removal of an inefficiency that would otherwise retard population group when producers are especially common: the rate-efficiency trade-off ensures that glucose isn't used as efficiently as it might be; the costly invertase production being uncoupled to sucrose levels provides an evident inefficiency. Population structure contributes to inefficiency by ensuring that some cells suffer costs while reaping poor benefits, owing the rate-efficiency trade-off and being exposed to the spike in glucose.

Given this, why is it that removal of just one inefficiency, leaving others, can restore the classical result? To see this consider that, while producers may be inefficient in some regards, they also diminish an inefficiency, as they convert inefficiently used sucrose into more efficiently used glucose. The question is not whether there are any inefficiencies but rather whether their net braking effects outweigh their net accelerating effects (removal of inefficiency). Importantly modification of just one cost/inefficiency has consequences for the others, potentially amplifying effects. For example, removal of cost has the direct consequence of faster growth of producers. However, as a knock-on effect, the population uses less sucrose, thus diminishing a further inefficiency. The effect is non-trivial, however, as it is further modulated by the rate-efficiency trade-offs.

## Discussion

While the dynamics of the situation are rather too complex to be captured fully by the above simple verbal explanations, these results do show that to understand the dynamics of social behavior in this circumstance it was helpful to have started by considering a model incorporating the details of the biology of our given circumstance. Moreover, the above can be seen as a successful case history for a modeling approach in which fitness is permitted to emerge from the underlying biochemistry, rather than being imposed or assumed. That this, in addition, captures new insight into co-operation dynamics suggests that our approach may be worth exploring in other contexts.

We should, however, also ask whether there are lessons from yeast that might be relevant elsewhere? In circumstances where growth is dependent upon a finite resource, a trade-off between the availability of publicly accessible resources and the efficiency with which they are used is likely to be commonplace. This is true for social scientific, economic, and evolutionary conditions. In the case of microbial metabolism, a trade-off between rate of resource uptake and efficiency will always exist because of thermodynamic constraints on metabolism [40]–[42], ensuring that resources will always be used less efficiently when they are abundant (see also [43]). Rate-efficiency trade-offs are also known to be a feature of human societies: food is wasted less when there is a famine. A rate-efficiency trade-off, we suggest, would be a valuable assumption for heuristic models to make (see also [44]).

What about yeast's inability to shut down invertase production immediately upon sucrose exhaustion? Does this have general relevance? To approach this issue, it is helpful to understand why yeast behave in the manner they do and whether similar constraints may apply elsewhere. That yeast invest in invertase production when such production isn't needed may not reflect an underlying inability of yeast to sense sucrose. Evidence suggests that yeast can sense sucrose through GPR1 [45],[46]. However, the same receptor is used to sense glucose. The problem may thus be a constraint whereby they cannot discriminate sucrose concentrations from glucose levels. Others sources of constraint-based informational inaccuracy would include an inability to directly detect the amount of invertase needed (i.e. absence of sucrose sensing) and, if they had a means to sense sucrose alone, error in any such assessment. Constraints of the above form may well be commonplace in non-conscious beings and in any circumstance where perfect information is lacking.

Alternatively invertase over-production may have an individual-level adaptive explanation, rather than a constraint-based explanation. That, for example, yeast secrete invertase in the absence of sucrose and glucose may be an adaptation to ensure a rapid response should sucrose become available. If an adaptive explanation for an uncoupling between the amount of co-operation needed and the amount offered is of some validity, then inappropriate levels of co-operation may well be commonplace. For the reasons above, we consider informational inaccuracy (or an uncoupling between level of co-operation needed and the level offered) to be of broad relevance. For similar reasons, we note a necessary caveat that, as with all experimental evolution, what we observe in the laboratory setting need not reflect what happens in the wild, i.e. in the context where the pattern of invertase production is expected in some manner to be optimal.

The assumption that the population is structured is likely to almost always be the case. Indeed, in the case of yeast, invertase is maintained in proximity of the producing cells [8]. There could thus be population structure as regards access to glucose, even if not as regards cell proximity, even in liquid culture. It was this that in part motivated our choice of vigorously shaken flasks for examination of the absence of population structure.

Another way to consider the generality of the result is to ask about the changes needed to the assumptions of simple snowdrift game to possibly recover our result. From the equation for population fitness, we can establish that for population fitness to be maximal when cheats and co-operators co-exist requires that S+T>2R. Why then might this be so? Our circumstance suggests a few possible generalizable extensions. First, the findings suggest the relevance of permitting different benefit terms for co-operators when meeting co-operators, for defectors meeting co-operators, and for co-operators meeting defectors. The last two are different not least because of the spatial structure ensuring different exposure to sucrose and glucose of the two cell types. The net effects on *S*, *T*, and *R* are not trivial. However, we can see why *T* might be increased while *R* is decreased. If both producers and non-producers see the same net amount of glucose, but the temporal dynamics are such that producers have this all in one brief shot, then we expect, from the rate-efficiency trade-off, that the benefit going to the producers would be lower than to the “cheats.” The former burn it up rapidly and inefficiently, while the latter use it more slowly and more economically. Such a trend would act to increase *T* and decrease *R* and *S*. However, simple extrapolation is not obviously warranted, as making the assumption that all cells see equal net amounts of glucose is hard to defend. Nonetheless, it is clear that *b* should not be considered a constant and that rate-efficiency trade-offs will have effects on the dynamics.

We should also not assume that the cost suffered by a co-operator when playing a fellow co-operator must be *c*/2, *c* being the cost suffered by a co-operator when playing a defector. This is equivalent to saying that the net cost of co-operation is not fixed. In our case, as invertase is produced dependent upon glucose levels and more of the sucrose is converted to glucose when everyone is a producer (otherwise sucrose is just consumed directly), the cost term for the co-operators against the co-operators may well be greater than *c*/2. If so, the difference in the cost terms in functions *R* and *S* relatively is reduced, effectively raising *S* and reducing *R* from the simple formulations.

The above all suggest rather general cases where it becomes more likely that *S*+*T*>2*R*. These game theoretical formulations are, however, too inexact to make precise specifications for our current context, as costs and benefit terms are both frequency dependent and the temporal dynamics of sugar usage seem also to be important. Indeed, in our example and perhaps in others, the language of “cheat” and “co-operator” obscures the reality. When the addition of more invertase producers reduces the fitness of all, it is hard to see invertase production as co-operation, even if it behaves in a more classical co-operative manner, benefitting all, when rare. We suggest that incorporation of both resource utilization efficiency (see e.g. [44]) and inaccuracy of information (see e.g. [47]) is likely to be both more realistic for multiple circumstances and potentially important to understand the dynamics of putatively co-operative social interactions under a broad range of circumstances.

## Materials and Methods

The experiments have been conducted using a yeast model system developed in Greig and Travisano [2]. It consists of two isogenic yeast strains, *SUC2* (a/α, *leu2/leu2*, *his5/his5*, *ura3/ura3*, *SUC2/SUC2*) and *suc2*, an isogenic diploid strain in which both copies of *SUC2* have been replaced by *KanMX*. *SUC2* secretes the enzyme invertase required to catalyze hydrolysis of sucrose into glucose and fructose and is therefore termed producer or co-operator, while the other strain *suc2* refrains from secreting invertase and is termed non-producer or cheat. For all experiments, yeast were grown in supplemented minimal medium (5 g/L ammonium sulphate, 1.7 g/L yeast nitrogen base, 50 mg/L uracil, 20 mg/L histidine, 50 mg/L leucine) containing agar (*16*g/L), sucrose, and glucose when necessary. All cultures were grown at a temperature of 30°C and liquid cultures were shaken using an orbital incubator (150 rpm). For further details of strains see [2],[7].

### Experimental Design A

*SUC2* and *suc2* were competed against each other for 24 h in 16 chemostats supplied with glucose-limited culture medium (0.8 g/L) incubated with continuous shaking and aeration. Dilution rate varied between 0.2 and 0.4 per hour. Using these conditions, glucose uptake rate is between 0.2 and 0.4 mmol/gram/hour [37], which induces the secretion of invertase in *SUC2* cells [18] so that invertase makes up approximately 0.1% of cell protein. Quantitative PCR and DNA extracted from samples taken from each chemostat before and after competition was used to measure the change in the abundance of *suc2* and *SUC2* during competition. Fitness was calculated as the ratio of population doublings during competition (w).

### Experimental Design B

Starter cultures of *SUC2* and *suc2* were grown up overnight in liquid YPD medium. Starter cultures were then diluted down 10^−4^ and each strain was inoculated onto 2 µM filters (Milipore, UK) that were placed on agar plates containing 100 g/L sucrose (10%) or 20 g/L sucrose (2%). Each strain was spread onto four filters on two agar plates of each sucrose concentration. One randomly selected filter was removed from each agar plate after 4 h, 24 h, 30 h, and 48 h. Filters were vortexed in sterile saline for approximately 30 s to form a cell suspension that was diluted down and plated out YPD plates to determine cell titre on each disk. Growth rate was calculated as the slope of population doublings against time during the exponential phase of growth. Results from the 10% and 2% sucrose plates were combined because growth rates were equal on these two media for both strains.

### Experimental Design C

We established competition cultures of a *SUC2* strain and a *suc2* strain that were grown up overnight in YPD broth. 20 mL agar plates, containing 20 g/L (2%) sucrose, were inoculated with 20 20 µL aliquots of competition cultures in a standardized 5 by 4 array. We consider two population structures. In the mixed population treatment, each aliquot on a plate consisted of the same mix of both *SUC2* and *suc2*. In the structured treatment each aliquot on a plate consisted of either *SUC2* or *suc2*. In total 12 competitions were carried out on mixed as well as structured plates. For the mixed treatment, starter cultures were mixed to form competition cultures where each aliquot consisted of a fixed proportion of *SUC2*, with the following cases being considered 20%, 40%, 60%, and 80% of *SUC2*. For the structured treatment, cases considered were 20%, 40%, 60%, and 80% of aliquots containing only *SUC2* while the rest of respective aliquots contained *suc2*. In this treatment, the position of *suc2* and *SUC2* aliquots on the array was randomized.

After all sugar was exhausted (population growth had ceased) the content of each agar plate was homogenized by washing cells off of the plate in 3 mL of sterile saline. The fitness of SUC2 and *suc2* was determined by quantitative PCR on DNA extracted from samples taken before and after competition. To estimate net titre, cells were serially diluted and spread on YPD plates to accurately determine cell numbers at the end of the experiment.

### Experimental Design D

The methods for this experiment were the same as for Experiment C with the following exceptions. 20 mL agar plates, containing 2% sucrose, were inoculated with single aliquot of competition culture containing 1.2×10^5^ cells evenly spread across the entire plate. We considered the cases where each aliquot contained SUC2 at an initial frequency of 0%, 20%, 40%, 60%, 80%, and 100%. After 2 d of incubation, the content of the agar plate was homogenized by washing off cells in 3 mL of sterile saline. To determine titre, we plated serial dilutions of this homogenized sample on YPD agar plates.

The titre data from Experiments C and D were subsequently normalized to maximum observed titre in each set-up before presenting in Figure 1.

### Experimental Design E

Starter cultures of *SUC2* and *suc2* were grown up for 2 d in liquid YPD medium, and then samples were diluted down and plated to yield single colonies on YPD agar, which were counted to determine the original cell density in the starter cultures. Mixtures of these starter cultures were made corresponding to 100%, 80%, 60%, 40%, 20%, and 0% by volume of the *SUC2* culture, and these were diluted 10-fold with sterile water. 13 µl of each of these diluted mixtures was pipetted onto the centre of 20 ml plates containing 0.1% or 0.01% sucrose. These plates were incubated for 7 d, then the patch of cells in the middle of each plate was cut out of the agar using a sterile scalpel and placed into 5 ml of sterile water in a capped test-tube. These test-tubes were vortexed vigorously to wash the yeast cells from the agar, and the resulting suspension was diluted down and plated out on YPD medium to determine the number of cells in each patch.

### Experimental Design F

For the experiment in liquid culture, 1.3 µl of each of the diluted cell mixtures, as described in Design E, was pipetted into 2 ml liquid 2% sucrose medium in 25 mm wide test-tubes. These were incubated for 2 d with shaking, before the cultures were diluted down and plated to determine the number of cells in each culture. The experiments were replicated three times.

### Quantitative PCR

DNA for use in quantitative PCR was extracted using a Wizard genomic DNA extraction kit (Promega, UK) as per the manufacturer's instructions. DNA was amplified using SYBR Green Master Mix (Applied Biosystems International) or TaqMan Universal PCR master mix (Applied Biosystems International), depending on whether or not a dual-labeled probe was used in the amplification reaction. Amplification reactions contained each primer at a concentration of 900 nM and a dual labeled probe (where appropriate) at a concentration of 62.5 nM. SYBR Green chemistry was used to detect the *SUC2* strain using forward (5′-CGATGATTTGACTAATTGGGAAGA-3′) and reverse primers (5′-CCAGAGAAAGCACCTGAATCGT-3′) that amplify a section of the *SUC2* gene. The *suc2* strain was detected using a dual-labeled probe (FAM-CGGGCAATCAGGTGCGACAATCTATC-TAM) that binds between forward (5′-GTATAAATGGGCTCGCGATAATG-3′) and reverse primers (5′-CATCGGGCTTCCCATACAAT-3′) of the *KanMX* gene. Amplifications were carried out in an ABI 7000 sequence detection under the following reaction conditions: 10 min at 95°C followed by 40 cycles or 95°C for 30 s followed by 60°C for 30 s. The relative copy number of a particular sequence in a given amplification reaction was determined by comparison with standard curves of DNA extracted from known reference strains. Each amplification reaction from a competition culture was carried out with at least 2- to 4-fold replication. Fitness was measured as ratio of doublings of the two strains during competition, such that a value of 1 represents equal competitive ability. Quantitative-PCR based methods have previously been used to measure fitness of yeast during competition, and preliminary experiments revealed that this protocol gives equivalent results to measuring the abundance of *SUC2* and *suc2* by plating samples of competition cultures on YPD and YPD supplemented with geneticin, which selects for the *suc2* strain.

## Supporting Information

Text S1 **Supplementary information incorporating Supplementary Methods 1 (parameter estimation), Supplementary Results 1 (alternative fitness measure), Supplementary Results 2 (comparison with Gore et al. 2009** [**8**] **), Supplementary Results 3 (experimental results for low sucrose), and Supplementary Results 4 (experimental results for homogeneous environments).**
(0.64 MB PDF)
