# Supplementary material for: A Mixture of “Cheats” and “Co-Operators” Can Enable Maximal Group Benefit
Source: PLoS Biol. 2010 Sep 14;8(9):e1000486. doi: 10.1371/journal.pbio.1000486 (PMC2939026; doi:10.1371/journal.pbio.1000486)
Supplement: Text S1 — Supplementary information incorporating Supplementary Methods 1 (parameter estimation), Supplementary Results 1 (alternative fitness measure), Supplementary Results 2 (comparison with Gore et al. 2009 [8] ), Supplementary Results 3 (experimental results for low sucrose), and Supplementary Results 4 (experimental results for homogeneous environments). (0.64 MB PDF) [file pbio.1000486.s001.pdf]

Supplementary methods 1: Parameter estimation

A number of estimates of the parameters in model (1) of the main text can be obtained from the literature. However these estimates are not necessarily obtained for the same laboratory yeast strains used in this study. In (Badotti et al. 2008) the authors measured the kinetics of active H+-sucrose symport activity in $1403-7$A strain and we use this data to define our sucrose uptake rate J^S as

$$J^S(S) = \left\{ \begin{array}{cc} 64 \cdot S, & 0 \leq S \leq 0.042 \\ 14.8 \cdot S + 2.1, & 0.042 < S \leq 0.43 \\ 2.04 \cdot S + 7.57, & 0.43 < S \leq 0.9 \\ 1.48 \cdot S + 8.08, & 0.9 < S \end{array} \right\}. \quad (1)$$

Here and throughout the manuscript we use the following conversion 1 g biomass $= 0.5$ g protein (personal communication with Karin Elbing). In Elbing et al., (2004) the authors estimated the relationship between invertase activity and glucose consumption rate using KOY.PK2-1C83 wild type strain and its various genetically manipulated derivatives. We use this data to estimate the invertase activity $inv(J^G)$ as

$$inv(J^S) = -2.3453 \cdot (J^G)^2 + 32.609 \cdot (J^G) + 77.109. \quad (2)$$

The estimates

$$V^G_{max} = 40 \frac{\text{mmol}}{\text{g protein } \times \text{ hour}}, \quad (3)$$

and

$$K^G_m = \left\{ \begin{array}{cc} 1.9\text{mmol/agar}, & \text{for 25 mL agar plate} \\ 1.52\text{mmol/agar}, & \text{for 20 mL agar plate} \end{array} \right\}, \quad (4)$$

are obtained from Otterstedt et al. (2004) which uses CEN.PK2-1C wild-type strain while the estimate

$$K^F_m = \left\{ \begin{array}{cc} 3.125\text{mmol/agar}, & \text{for 25 mL agar plate} \\ 2.5\text{mmol/agar}, & \text{for 20 mL agar plate} \end{array} \right\}, \quad (5)$$

was obtained from Reifenberger et al. (1997). The remaining parameters of the model (1) in the main text , for which there are no estimates already available in the literature, are estimated using simple growth experiments described below.

0.1 The growth experiments

Yeast cells (producers or non-producers) were initially distributed in two identical patches per 25mL agar plate, 5cm apart. The patches were initially made up of 5 μl of a liquid culture grown overnight in YFPD broth. The assays were replicated 6 times. The initial and final cell densities were measured in CFU/patch which is converted into g protein/patch in table below using the assumption that 1mg protein $= 33 * 10^6$ cells. This assumption is used throughout the manuscript.

	Cell type	Carbon source	Initial cell biomass g protein/per patch	Length of experiment	Final cell biomass g protein/per patch (replicate average)
Experiment 1a	n-p	sucrose 0.2%	$9.737 * 10^{-6}$ (replicate average)	4 days	0.00215
Experiment 1b	n-p	sucrose 2%	$9.737 * 10^{-6}$ (replicate average)	4 days	0.0083
Experiment 2a	n-p	glucose 0.2%	$9.17 * 10^{-6}$	2 days	0.0029
Experiment 2b	n-p	glucose 2%	$9.17 * 10^{-6}$	2 days	0.0047
Experiment 3a	n-p	fructose 0.2%	$9.17 * 10^{-6}$	2 days	0.0025
Experiment 3b	n-p	fructose 2%	$9.17 * 10^{-6}$	2 days	0.0053
Experiment 4a	p	fructose 0.2%	$9.4 * 10^{-6}$	2 days	0.0023
Experiment 4b	p	fructose 2%	$9.4 * 10^{-6}$	2 days	0.005
Experiment 5a	n-p	glucose 0.2% & fructose 0.2%	$9.17 * 10^{-6}$	2 days	0.0032
Experiment 5b	n-p	glucose 2% & fructose 2%	$9.17 * 10^{-6}$	2 days	0.0043
Experiment 6a	p	sucrose 0.2%	$7.663 * 10^{-6}$	4 days	0.0071
Experiment 6b	p	sucrose 2%	$7.663 * 10^{-6}$	4 days	0.011

0.1.1 Data fitting

Throughout the manuscript data fitting is conducted in the following way. The Experiments $1 - 6$ have been set up with two patches on an agar plate each containing the same amount and the same type of cells. The initial and the final cell density in the experiment are recorded per patch. Therefore the initial cell density in the numerical simulation of models (6 – 9) and model (13) is the same as initial cell density in one experimental patch. Mathematical models of growth kinetics (6 – 9) described below are developed at a single patch level. The concentration of carbon sources in the experiment is measured per agar plate (which contains two identical patches) and therefore the initial concentration of carbon resources in the numerical simulation is half of the experimental concentration measured per agar. We run numerical simulations for the same length of time experiments are conducted and find the value of the free parameter that gives rise to the same final cell density in a patch as in the experiment.

0.2 Estimating the rate-efficiency trade-off of H^+ - symport

The growth of non-producers (N_n) on sucrose can be represented by a simple model

$$\frac{dS}{dt} = -J^S \cdot N_n, \quad (6a)$$
$$\frac{dN_n}{dt} = n_e^S \cdot J^S \cdot N_n. \quad (6b)$$

Having fixed the sucrose uptake rate J^S as in (1), the efficiency of sucrose pathway n_e^S as a function of J^S is estimated from a simple growth experiment of non-producers on sucrose (Experiment 1). We assume the following form of the efficiency function:

$$n_e^S(x) = \begin{Bmatrix} a_1, & 0 \leq x \leq \text{maximal uptake rate at 0.2\% sucrose} \\ b_1, & x > \text{maximal uptake rate at 0.2\% sucrose} \end{Bmatrix}.$$

Next we estimate $a_1 = 0.0295\ \frac{\text{g protein}}{\text{mM sucrose}}$ by fitting the model (6) to final population data in Experiment 1a, and we estimate $b_1 = 0.0094\ \frac{\text{g protein}}{\text{mM sucrose}}$ by fitting the same model to the final population data in Experiment 1b.

0.3 Estimating the rate-efficiency trade-off of hexose transport

The growth of non-producers (N_n) on glucose can be represented by a simple model

$$\frac{dG}{dt} = -J^G \cdot N_n, \quad (7a)$$
$$\frac{dN_n}{dt} = n_e^{Hxt}(J^G) \cdot J^G \cdot N_n, \quad (7b)$$

where $J^G = \frac{V_{max}^G \cdot G}{K_m^G + G}$ which is obtained by setting $\frac{F}{K_c^G} = 0$ in (10) as there is no fructose in the environment and hence no competition for the hexose transporters.

Since V_{max}^G and K_m^G are fixed as in (3) and (4) respectively, the efficiency of hexose pathway n_e^{Hxt} as a function of the uptake of hexose resources (in this case glucose only) is estimated from a simple growth experiment of non-producers on glucose. We use data in Weusthuis et al. (1994) to define the following form of the rate-efficiency function illustrated in Figure 1 . Note, we do not use the values obtained in (Weusthuis et al., 1994) for the rate-efficiency function as these have been obtained in the chemostat. As pointed out in van Dijken et al. (2000) biomass yield estimates are found to be lower in chemostats than when obtained from agar plates. Instead we rely on (Weusthuis et al., 1994) only for the form of the function n_e^{Hxt} but use agar plate growth experiments (Experiments 2) to estimate the precise values in n_e^{Hxt}.

Fitting the model (7) to the final population data in Experiment $2a$ we estimate $a_2 = 0.048\ \frac{\text{g protein}}{\text{mM sucrose}}$ while fitting the same model to the final population data in Experiment $2b$ we arrive at $b_2 = 0.00632$

Figure 1: Rate-efficiency trade-off n_e^{Hxt}. The parameters x_1, x_2 and x_3 denote the maximal uptake rate at 0.2%, 2% and 4% glucose respectively.

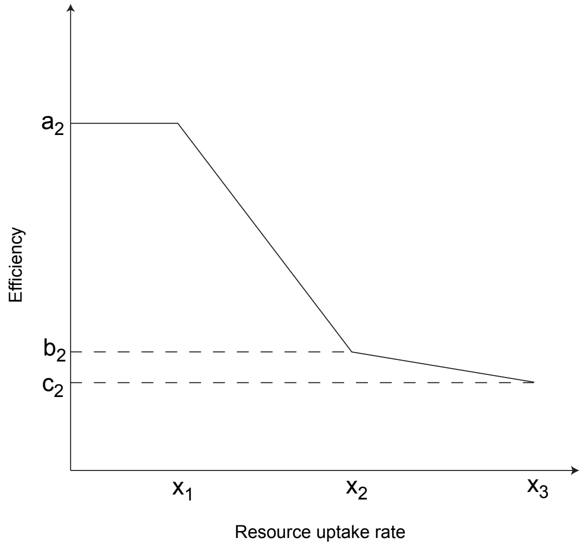

$\frac{\text{g protein}}{\text{mM sucrose}}$. The parameter c_2 is only needed further on in the manuscript when fitting the model to Experiment 5b, and we take $c_2 = 0.001$ as some small perturbation of b_2. Note that given the competition between glucose and fructose for hexose transporters, and the spatial experimental setup (see Methods, main text) the uptake rate of glucose and fructose is never greater than x_2 in the fully spatial model (2) in the main text.

0.4 Estimating the maximal uptake rate of fructose

The growth of non-producers on fructose can be represented by a simple model

$$\frac{dF}{dt} = -J^F \cdot N_n, \tag{8a}$$

$$\frac{dN_n}{dt} = n_e^{Hxt}(J^F) \cdot J^F \cdot N_n, \tag{8b}$$

where $J^F = \frac{V_{max}^F \cdot F}{K_m^F + F}$ which is obtained by setting $\frac{G}{K_c^F} = 0$ in (11) as there is no glucose in the environment and hence no competition for the hexose transporters. Since K_m^F is fixed in (5) and n_e^{Hxt} has been estimated in the previous section, the maximal fructose uptake rate V_{max}^F is the only free parameter. In this case we have two experimental values for the final cell densities one for growth on 2% fructose and the other for growth on 0.2% fructose (Experiments $3a$ and $3b$, respectively). As our model has only one free parameter V_{max}^F, we fit the model (8) to Experiment $3b$ and arrive at $V_{max}^F = 54.93\ \frac{\text{mmol}}{\text{g protein} \times \text{hour}}$. Next we check whether using the obtained value of V_{max}^F in the model (8) we can correctly predict the outcome of Experiment $3a$ and find that we can.

0.5 Estimating the cost of invertase production

Experiment 7
We competed producer and non-producer strains in glucose-limited chemostats under conditions that induce the secretion of invertase without any possible benefit of invertase secretion. The two strains were competed against each other for 24 hours in 16 chemostats supplied with glucose-limited culture medium (0.8 g/L) incubated with continuous shaking and aeration. Dilution rate varied between 0.2 and 0.4 per hour. Under these conditions, glucose uptake rate is between 0.2 and 0.4 mmol.gram^{-1}.hour^{-1} (Weusthuis et al., 1994), which induces the secretion of invertase in producer cells (Elbing et al., 2004) so that invertase makes up approximately 0.1% of total cell protein. Quantitative PCR on DNA extracted from samples taken from each chemostat before and after competition was used to measure the changes in the abundance of producers and non-producers during competition. Fitness was calculated as the ratio of population doublings during competition (w). In this experiment it was found that the non-producers enjoy a small, but detectable, fitness advantage (mean $w = 1.04$, s.e= 0.014, n= 15, $t_{14} = 2.72$, $P = 0.016$).

Remembering that the cost of invertase production is defined as

$$c^{Inv} = inv(J^G)U^{Inv},$$

where U^{Inv} denotes the unit cost of invertase which is a function of invertase activity $inv(J^G)$. Motivated by Dong et al., 1995 and taking into account the results of Experiments 7 we arrive at

$$U^{Inv} = 0.00054e^{0.000001inv(J^G)}.$$

0.6 Estimating the hexose transporters competition functions for glucose (K_c^G) and fructose (K_c^F)

The competition functions are estimated using the following two models. The growth of non-producers on glucose and fructose can be represented by a model

$$\frac{dG}{dt} = -J^G \cdot N_n, \tag{9a}$$

$$\frac{dF}{dt} = -J^F \cdot N_n, \tag{9b}$$

$$\frac{dN_n}{dt} = n_e^{Hxt}(J^G + J^F) \cdot (J^G + J^F) \cdot N_n, \tag{9c}$$

where J^G and J^F are defined as

$$J^G = \frac{V_{max}^G \cdot G}{K_m^G \cdot \left(1 + \frac{F}{K_c^G}\right) + G} \tag{10}$$

$$J^F = \frac{V^F_{max} \cdot F}{K^F_m \cdot \left(1 + \frac{G}{K^F_c}\right) + F}. \tag{11}$$

Similarly, the growth of producers on sucrose can be represented by a model

$$\frac{dS}{dt} = -J^S \cdot N_p - Inv \cdot N_p, \tag{12a}$$

$$\frac{dG}{dt} = -J^G \cdot N_p + Inv \cdot N_p, \tag{12b}$$

$$\frac{dF}{dt} = -J^F \cdot N_p + Inv \cdot N_p, \tag{12c}$$

$$\frac{dN_p}{dt} = (1 - c^{Inv}) \cdot (n_e^{Hxt}(J^G + J^F) \cdot (J^G + J^F) + n_e^S \cdot J^S) \cdot N_p. \tag{12d}$$

The free parameters in the above models (9) and (12) are hexose competition functions K_c^G, K_c^F the first one is the function of glucose concentration while the second one is a function of fructose concentration in the environment. We use Experiments 5 and 6 to estimate these functions and in view of the experimental setup we assume the following forms of the competition functions:

$$K_c^G(x) = \left\{ \begin{array}{ll} a_3, & 0 \leq x \leq y_1^G \\ b_3, & y_1^G \leq x \leq y_2^G \\ c_3, & x > y_2^G \end{array} \right\},$$

and

$$K_c^F(x) = \left\{ \begin{array}{ll} a_4, & 0 \leq x \leq y_1^F \\ b_4, & y_1^F \leq x \leq y_2^F \\ c_4, & x > y_2^F \end{array} \right\}.$$

The constant y_1^G (y_1^F) denotes the 0.2% glucose (fructose) concentration while $y_2^G(y_2^F)$ denotes the glucose (fructose) concentration created from degradation of 2% sucrose. Fitting the model (9) and ((12)) to final population data in Experiment 5a and 6a we estimate $a_3 = 0.67$ mM and $a_4 = 0.05$ mM. Fitting the model (12) to final population data in Experiment 6b we estimate $b_3 = 0.03$ and $b_4 = 0.0088$ mM. Finally fitting the model (9) to the final population data in Experiment 5b we estimate $c_3 = 0.132$ mM and $c_4 = 0.0174$ mM. In each fitting procedure we have two free parameters and one experimental data point so in our choice of the estimates we use the following criteria: glucose is used preferentially over fructose and the sum of the maximal glucose and fructose uptake rate should be such that there is a rate-efficiency trade-off of hexose transporter, in other words $x_1 < max\{J^G + J^F\} \leq x_2$, where x_1 and x_2 are defined in Figure 1.

0.7 Estimating resource movement parameter D

Experiment 8: Yeast cells (producers or non-producers) were initially distributed in two identical patches per 25mL agar plate, 5cm apart. The patches were initially made up of 5 μl of a liquid culture grown overnight in YFPD broth. The assays were replicated 6 times. Note, the initial and final cell densities were measured in CFU/patch which is converted into g protein/patch in table below using the assumption that 1mg protein $=$ $33 * 10^6$ cells.

Carbon source	Initial cell biomass producers g protein/patch 1	Initial cell biomass of non-producers g protein/patch 2	Length of experiment	Final cell biomass of producers g protein/patch 1	Final cell biomass of non-producers g protein/patch 2
0.2% sucrose	$1.28 * 10^{-5}$	$9.7 * 10^{-6}$	4 days	0.0063	0.0035

Note that the distance between patches does not alter the outcome (data not shown). Now consider $m = 0$ in the model (2) of the main text which consists of two spatially segregated regions, the first one containing producers only and the second one containing non-producers only. As mentioned before the yeast cells are non-motile but the resources can move between two regions at a diffusion rate D. Due to the molecule size we assume that the rate of movement of sucrose is twice as slow as that of glucose or fructose. In this case the growth of producers and non-producers growing on sucrose is modeled by

$$\frac{dS_1}{dt} = -J^{S_1} \cdot N_{1p} - Inv \cdot N_{1p} + \frac{D}{2} \cdot (S_2 - S_1), \tag{13a}$$

$$\frac{dS_2}{dt} = -J^{S_2} \cdot N_{2n} + \frac{D}{2} \cdot (S_1 - S_2), \tag{13b}$$

$$\frac{dG_1}{dt} = -J^{G_1} \cdot N_{1p} + Inv \cdot N_{1p} + D \cdot (G_2 - G_1), \tag{13c}$$

$$\frac{dG_2}{dt} = -J^{G_2} \cdot N_{2n} + D \cdot (G_1 - G_2), \tag{13d}$$

$$\frac{dF_1}{dt} = -J^{F_1} \cdot N_{1p} + Inv \cdot N_{1p} + D \cdot (F_2 - F_1), \tag{13e}$$

$$\frac{dF_2}{dt} = -J^{F_2} \cdot N_{2n} + D \cdot (F_1 - F_2), \tag{13f}$$

$$\frac{dN_{1p}}{dt} = (1 - c^{Inv}) \cdot (n_e^{Hxt}(J^{G_1} + J^{F_1}) \cdot (J^{G_1} + J^{F_1}) + n_e^{S_1} \cdot J^{S_1}) \cdot N_{1p}, \tag{13g}$$

$$\frac{dN_{2n}}{dt} = (n_e^{Hxt}(J^{G_2} + J^{F_2}) \cdot (J^{G_2} + J^{F_2}) + n_e^{S_2} \cdot J^{S_2}) \cdot N_{2n}, \tag{13h}$$

where the subscript $i = 1, 2$ denotes patch number. All the parameters in the above model (13) are fixed apart from D and fitting the model to the final population data in Experiment 8 we arrive at $D = 0.007$.

Supplementary results 1: Alternative fitness measure

Rather than using the total titre as a measure of population fitness, we use the population growth rate as defined by Hegreness et al., 2008:

$$growth\ rate\ = \frac{(N_{end} - N_{start})/2}{t_{mid}},$$

where N_{start} denotes the initial population size and N_{end} denotes the population size just after the resources have been exhausted. The parameter t_{mid} is the time at which the population size reaches $(\frac{N_{end}-N_{start}}{2})$.

1a

Expected population growth rate as a function of initial producer frequency.

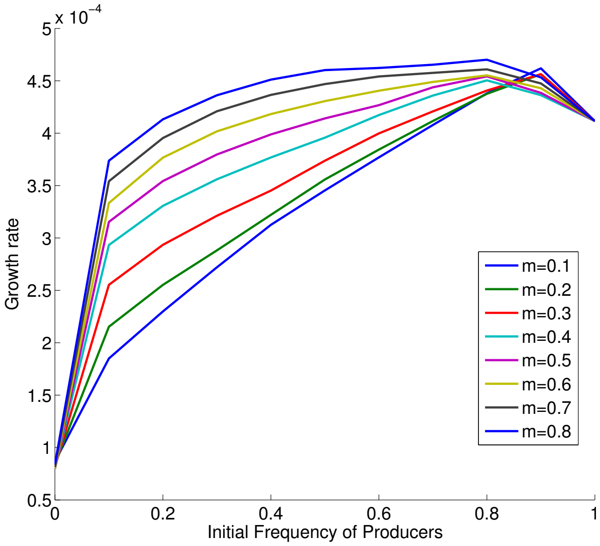

1b

Expected population growth rate as a function of initial producer frequency in the absence of rate-efficiency trade-off.

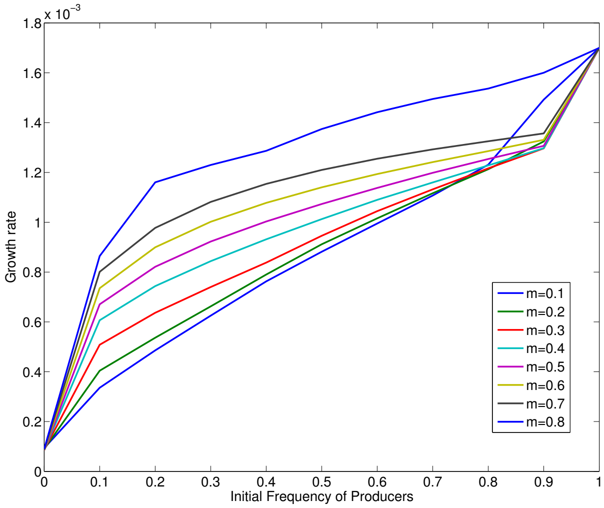

1c

Expected population growth rate as a function of initial producer frequency when cost of invertase is lowered.

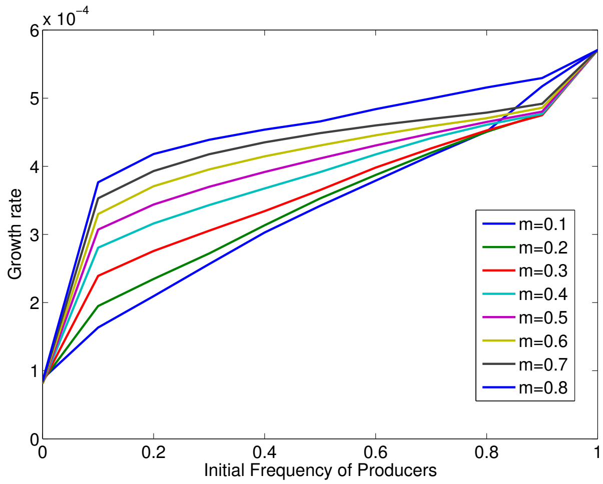

1d

Expected population growth rate as a function of initial producer frequency when invertase production matches sucrose levels (perfect information).

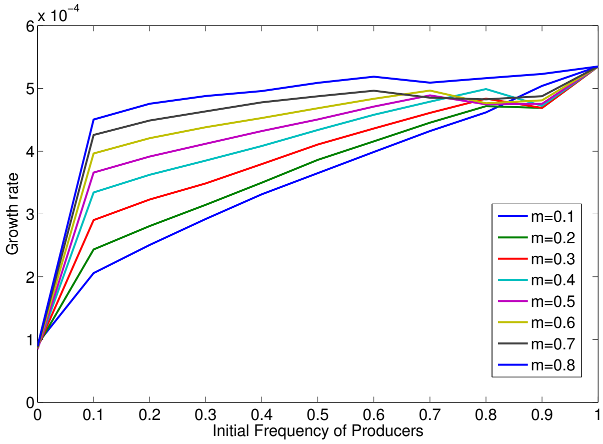

1e

Expected population growth rate as a function of initial producer frequency in well-mixed environments.

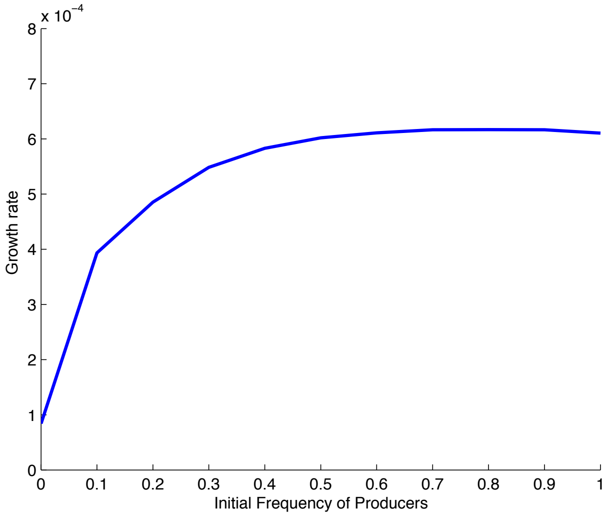

Supplementary results 2: Comparison with Gore et al. 2009

2a

Results	Gore et al. (2009) experiments	Gore et al. (2009) theory	Our experiments	Our theory
Glucose capture capture efficiency	1%	included as a constant	not measured	function of spatial structure, and resource levels (i.e glucose, fructose, sucrose)
Cost of invertase production	2.5%	included as a constant	4%	function of invertase expression (depends on glucose levels)
Growth assumptions		power law function of glucose levels		non-linear function of glucose fructose and sucrose levels (with rate-efficiency trade-off)
Fraction of producers at equilibrium	30% (flask cultures, 5% sucrose)	3.2% (for parameters estimated experimentally in Gore et al. 2009)	48% (structured agar plate, 2% sucrose) 36% (unstructured agar plate, 2% sucrose)	$[42\% - 55\%]$ (low mixing - equiv. to structured agar plate) $[31\% - 39\%]$ (high mixing - equiv. to unstructured agar plate)
Effect of increasing cooperator frequency on population size	not measured	n/a (densities not modeled)	1. at low frequencies total population increases 2. at high frequencies total population decreases	1. at low frequencies total population increases 2. at high frequencies total population decreases
Effect of increasing cooperator frequencies on growth rate	1. at low frequencies growth rate increases 2. at high frequencies not measured	1. at low frequencies growth rate increases 2. at high frequencies growth rate increases	not measured	1. at low frequencies growth rate increases 2. at high frequencies growth rate decreases
Effects of increasing cost of cooperation	1. equilibrium frequency of producers decreases 2. mean growth rate decreases	1. in agreement with experiments 2. in agreement with experiments	not measured	1. in agreement with experiments (Suppl. result 2b) 2. in agreement with experiments (Suppl. result 2c)
Effects of increasing cell density	cell grow faster at high than at low cell densities	n/a (densities not modeled)	not measured	in agreement with experiments (Suppl. result 2d)

2b

Relative producer fitness as a function of initial frequency in theory for the low cost of invertase production described in Figure 4c of the main text. The model predicts that for $m \in [0.5, 0.8]$, an equilibrium frequency is in the range $0.459 - 0.540$ while for $m \in [0.4, 0.1]$ the equilibrium frequency is in the range $0.569 - 0.634$ demonstrating that lowering the cost of invertase production leads to an increase in the equilibrium frequency of producers.

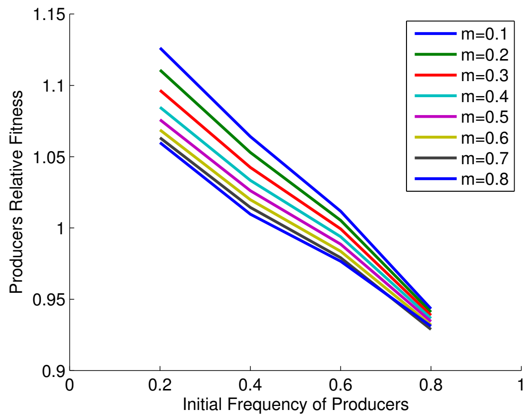

2c

Expected population growth rate as a function of initial producer frequency for varying cost of invertase production. The term "high cost" denotes the experimentally estimated cost used throughout this manuscript while the term "low cost" denotes the reduction in the cost of invertase production described in Figure 4c of the main manuscript. In both cases $m = 0.8$ representing high degree of mixing.

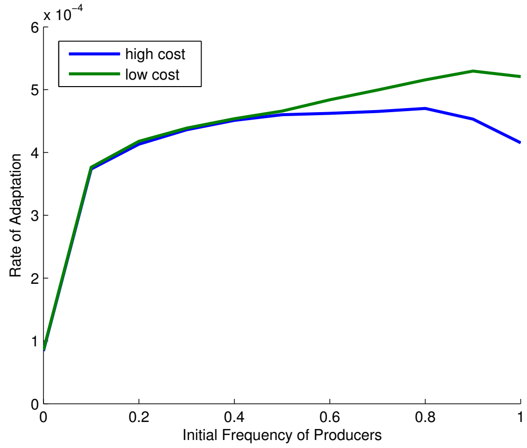

2d

Expected population growth rate as a function of initial producer frequency for varying total initial cell densities. Low density represents 7.27×10^{-6} g protein; intermediate density represents 1.454×10^{-5} g protein while high density represents 2.181×10^{-5} g protein. In all three cases $m = 0.8$ representing high degree of mixing.

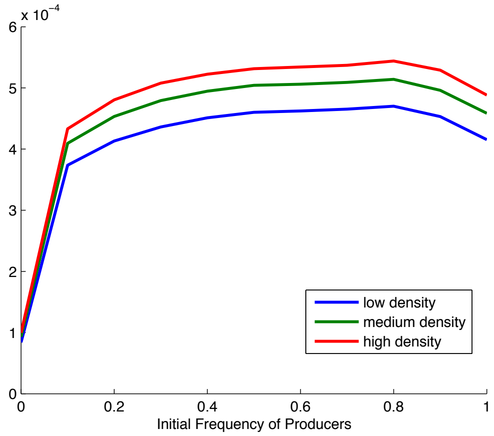

Supplementary results 3: Experimental results for low sucrose

3a

Experimental data for final population size as a function of initial producer frequency, derived from the experiment E in the main text with 0.01% initial sucrose.

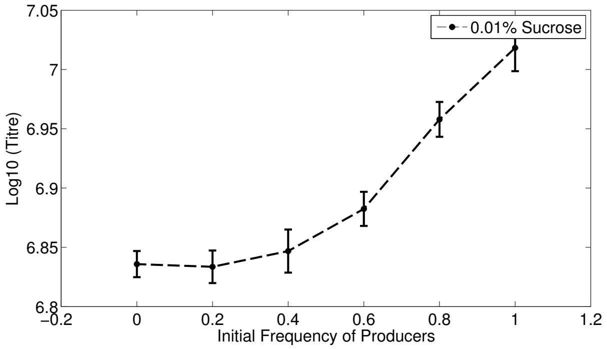

3b

Experimental data for final population size as a function of initial producer frequency, derived from the experiment E in the main text with 0.1% initial sucrose.

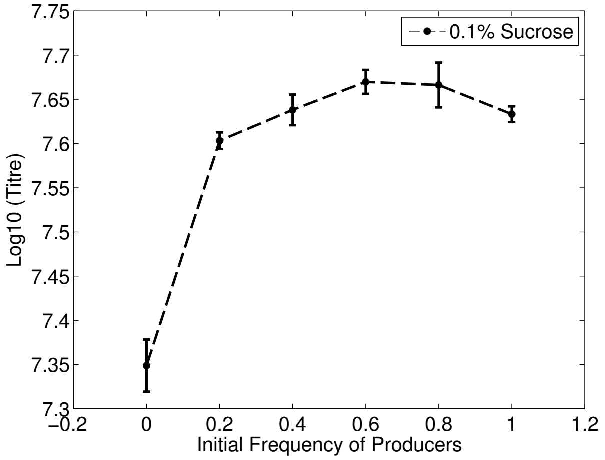

Supplementary results 4: Experimental results for homogeneous environments

Experimental data for final population size as a function of initial producer frequency, derived from the experiment F with 2% initial sucrose.

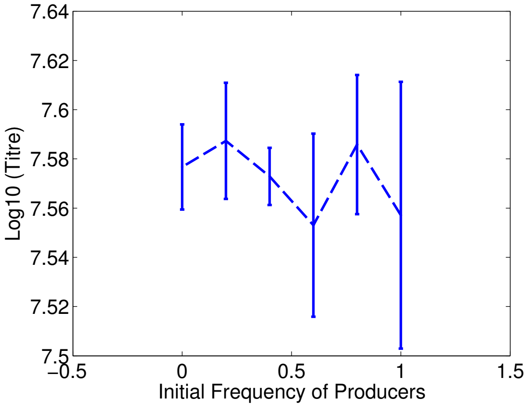

Theoretical results show that this observation is insensitive to sucrose concentration and that titre plots for 2% - 0.002% initial sucrose always approximate to a flat line.

References

Badotti F., Dario M. G., Alves Jr S. L., Luiza M., Cordioli A., Miletti L. C., de Araujo P. S. and Stambuk B. U. (2008) Switching the mode of sucrose utilization by *Saccharomyces cerevisiae Microbial Cell Factories* **7**(4).

Dong H., Nilsson L., and Kurland C.G. (1995). Gratuitous overexpression of genes in Escherichia coli leads to growth inhibition and ribosome destruction. *Journal of Bacteriology* **177**, 14971504.

Elbing K., Ståhlberg A., Boles E., Hohmann S., and Gustafsson L. (2004) Transcriptional responses to glucose at different glycolytic rates in *Saccharomyces cerevisiae European Journal of Biochemistry* **271**, 4855-4864.

Gore J., Youk H., and van Oudenaarden A. (2009) Snowdrift game dynamics and facultative cheating in yeast. *Nature* **459** 253-256.

Hegreness M., Shoresh N., Damian D., Hartl D., and Kishony R. (2008) Accelerated evolution of resistance in multidrug environments. *PNAS* **105** 1397713981.

Ottersteadt K., Larsson C., Bill R. M., Ståhlberg A., Boles E., Hohmann S., and Gustafsson L. (2004) Switching the mode of metabolism in the yeast *Saccharomyces cerevisiae EMBO reports* **5**(5), 532-537.

Reifenberger E., Boles E. and Ciriacy M. (1997) Kinetic characterization of individual hexose transporters of *Saccharomyces cerevisiae* and their relation to the triggering mechanisms of glucose repression. *European Journal of Biochemistry* **245** 324-333.

van Dijken J. P., Bauer J., Brambilla L., Duboc P., Francois J. M., Gancedo C., Giuseppin M. L. F., Heijnen J. J., Hoare M., Lange H. C., Madden E. A., Niederberger P., Nielsen J., Parrou J. L., Petit T., Porro D., Reuss M., van Riel N., Rizzi M., Steensma H. Y., Verrips C. T., Vindelov J. and Pronk J. T. (2000) An interlaboratory comparison of physiological and genetic properties of four *Saccharomyces cerevisiae* strains *Enzyme and Microbial Technology* **26**, 706-714.

Weusthuis R. A., Pronk J. T., van den Broek P. J. A. M. and van Dijken J. P. (1994) Chemostat Cultivation as a Tool for Studies on Sugar Transport in Yeast. *Microbiological Reviews* **58**(4), 616-630.
